# Association between two single base polymorphisms of intercellular adhesion molecule 1 gene and inflammatory bowel disease 

**Published:** 2016

**Authors:** Manijeh Habibi, Nosratllah Naderi, Alma Farnood, Hedieh Balaii, Tahereh Dadaei, Shohreh Almasi, Homayoun Zojaji, Hamid Asadzadeh Aghdae, Mohammad Reza Zali

**Affiliations:** 1 *Basic and Molecular Epidemiology of Gastrointestinal Disorders Research Center, Research Institute for Gastroenterology and Liver Diseases, Shahid Beheshti University of Medical Sciences, Tehran, Iran*; 2 *Research Institute for Gastroenterology and Liver Diseases, Shahid Beheshti University of Medical Sciences, Tehran, Iran*; 3 *Ayatollah Musavi Hospital, Zanjan University of Medical Sciences, Tehran, Iran*

**Keywords:** Inflammatory bowel disease, ICAM1 gene, Polymorphism, Crohn’s disease

## Abstract

**Aim:**

The present study evaluated the association between G241R and K469E polymorphisms of intercellular adhesion molecule 1 gene and inflammatory bowel disease in Iranian population.

**Background:**

Inflammatory bowel disease including ulcerative colitis and Crohn’s disease, is a chronic idiopathic inflammatory disease of the gastrointestinal tract. There are two single base polymorphisms of intercellular adhesion molecule 1gene, G241R and K469E, reported to be associated with inflammatory disorders.

**Patients and methods:**

In this case-control study, 156 inflammatory bowel disease patients (110 ulcerative colitis and 46 Crohn’s disease patients) and 131 healthy controls were enrolled. Two polymorphisms of intercellular adhesion molecule 1 gene, including G241R and K469E, were assessed by polymerase chain reaction followed by restriction fragment length polymorphism.

**Results:**

The E469 allele of K469E polymorphism was significantly more frequent in Crohn’s disease patients compared to controls (P< 0.05, OR= 1.83; 95% CI: 1.13 to 2.96). The mutant homozygote genotype of K469E polymorphism (E/E) was also significantly more frequent in Crohn’s disease patients compared to controls (P< 0.05, OR= 4.23; 95% CI: 1.42 to 12.59). No difference was observed in the frequency of K469E polymorphism among ulcerative colitis patients compared to controls. There were no significant differences in genotype and allele frequencies of G241R polymorphism among ulcerative colitis and Crohn’s disease patients compared to control subjects.

**Conclusion:**

According to our findings, K469E polymorphism of intercellular adhesion molecule 1 gene may probably participate in the pathogenesis of Crohn’s disease in Iran.

## Introduction

 Inflammatory bowel disease (IBD), a chronic inflammatory disorder of the gastrointestinal tract (GI), consists of ulcerative colitis (UC) and Crohn’s disease (CD) (1). Crohn’s disease affects all layers of the intestinal wall and may cause damage to any part of the gastrointestinal tract. However, most frequently targets the terminal ileum and colon. By contrast, the typical inflammation of ulcerative colitis is confined to the mucosa; it begins in the rectum and extends to a variable extent in continuity but does not affect the small intestine (2). The precise etiology of IBD is still unknown, but infectious agents as well as genetic background are proposed to contribute to the pathogenesis of disease. Genetic alterations may cause uncontrolled host defense against luminal antigens and is triggered by other environmental factors. The role of genetic factors in IBD was first suggested by epidemiologic data and confirmed by familial aggregation studies, mostly in CD patients (3). Microsatellite markers were then used to identify the areas of linkage with IBD in the whole genome. Among several loci identified to be linked with IBD, a significant association was recognized between IBD and chromosome 19p13 using linkage analysis (4). 

This locus contains intercellular adhesion molecule (ICAM)-1 gene, which encodes ICAM-1 protein. The ICAM-1 protein is a well-characterized surface glycoprotein, which is expressed on thymic epithelial cells, vascular endothelial cells, macrophages, and activated lymphocytes. It promotes the initial interaction between macrophages and T cells during immune activation (5). 

The ICAM-1 protein is also involved in various leukocyte functions, including antigen presentation, and extravasation into lymphoid and inflamed non-lymphoid tissues (2). It is documented that ICAM-1 protein is up regulated in the inflamed mucosa of IBD patients, which has a critical role in regulating the leukocyte localization in inflammatory parts. Thus, it may be an important mechanism in regulation of inflammatory responses in IBD (6-8). 

Two single-base polymorphisms of the ICAM-1 gene have been described, G241R and K469E. The G241R polymorphism at position 241 of the coding region of exon 4, results in Arginine (R) being replaced by a glycine (G) at 241^th^ amino acid (4). The K469E polymorphism in exon 6 of this gene results in a glutamate (E) being positioned by a lysine (K) at 469^th^ amino acid in the C-terminus of immunoglobulin domain 5. This domain has been reported to play a role for adhesion of dendritic cells and B-cells, thus it is the ICAM1 immunomodulant epitope (7, 8). It also has been demonstrated that K469E polymorphism is associated with CD in Caucasian populations (6). Considering the overall data about these polymorphisms; different associations were observed between these polymorphisms and IBD in various parts of the world (2, 9, 10). 

Ethnically defined population studies seem necessary to investigate the genetic role of ICAM-1 in IBD pathogenesis. To fulfill this goal, we examined the association of two ICAM-1 gene polymorphisms (G241R and K469E) with IBD in Iranian patients. 

## Patients and Methods

In this case-control designed study, 156 IBD patients, including 110 patients with UC, 46 patients with CD and 131 sex- and age-matched healthy controls from Iranian origin, were enrolled. Patients were referred to a tertiary GI center (Taleghani hospital, Tehran, Iran) in a 4-year period (2008-2011). Diagnosis of IBD was based on endoscopic, radiologic, and histo-pathologic findings (11, 12) by an expert gastroenterologist. Control subjects were selected from healthy individuals without any GI symptoms or positive family or personal history of GI disorders. A complete clinical questionnaire was filled for each patient at admission. The study protocol was approved by the ethics committee of the Research Center for Gastroenterology and Liver Diseases (Shahid Beheshti University of Medical Sciences, Tehran, Iran). A written informed consent was obtained from each participant.

Genomic DNA was extracted from whole blood leukocytes using the standard phenol chloroform method (13). 

Polymerase chain reaction (PCR) and restriction length polymorphism (RFLP) methods were used to detect G241R and K469E polymorphisms of ICAM-1 genes. 

A pair of primers was utilized for PCR of each polymorphism as follows (14): 5′-CCGTGGTCTGTTCCCTGTAC-3′ as forward primer and 5′-GAAGGAGTCGTTGCCATAGG-3′ as the reverse primer for G241R polymorphism and 5′-TTCCCAGCAGACTCCAATGT-3′ as forward and 5′-GGATACAACAGGCG -3′ as the reverse primer for K469E polymorphism (15).

Polymerase chain reactions (PCRs) were performed in a 25-μl volume containing 10 mM Tris–HCl, pH 8.8, 1.5 mM MgCl2, 50 mM KCl, 250 mM dNTPs, a 0.50-mM concentration of each primer, 2 mM of Taq DNA polymerase (Fermentas, Germany), and 200 ng of genomic DNA. The PCR condition for both fragments was as follows: initial 5 min at 94°C, followed by 35 cycles, consisting of denaturation for 30 sec at 94°C, annealing for 30 sec at 58°C, for R241 and 60°C for K469E and extension for 30 sec at 72°C. Polymerase chain reaction was performed with a personal thermal cycler (Eppendorf, Germany). 

**Figure 1:**
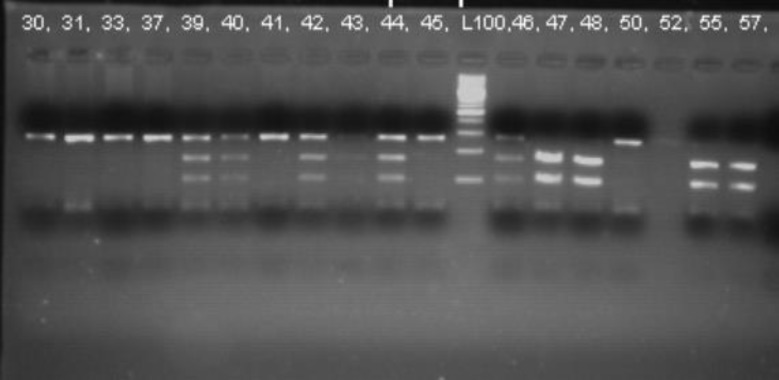
The digested fragments of K469E polymorphism of 17 patients with CD on agarose gel after digestion with BstUI restriction enzyme. L100, ladder 100 bp

Amplified segments were analyzed by electrophoresis on 3.5% agarose gel, stained with ethidium bromide and observed under ultraviolet light (Figure 1). The PCR product G241R (120 bp in size) was digested at 58°C for 12 h with 0.5U of BsrGI restriction enzyme (Fermentas, Germany), resulting in the following fragments: 20 and 90 bps. The PCR product for K469E (268 bp in size) was digested at 37°C for 12 h with 0.5U BstUI restriction enzyme (Fermentas, Germany) resulting in the following fragments: 170 and 98 bps. Some results were randomly genotyped in duplicate and 10% of the results were confirmed by DNA sequencing (all results were concordant). The genotype-phenotype correlation was analyzed.

Genotype and allele frequencies of G241R and K469E polymorphisms were calculated from observed genotype counts. Genotype frequencies for each polymorphism were all in the Hardy–Weinberg equilibrium among cases and controls.

**Table 1:** Allele frequencies of G241R and K469E polymorphisms of ICAM-1 gene in Iranian CD patients compared to healthy controls

SNP	Allele	CD(n=46)	p-value	OR (95%CI)	Control (n=131)
G241R	G	61(66.3)	0.05	1.68 (0.47-0.98)	202 (77.1)
	R	31(33.7)			60 (22.9)
K469E	K	41 (44.6)	0.015*	0.73 (0.58-0.92)	156 (59.5)
	E	51 (55.4)			106 (40.5)

**Table 2:** Allele frequencies of G241R and K469E polymorphisms of ICAM-1 gene in Iranian UC patients compared to healthy controls

SNP	Allele	UC(n=110)	p-value	OR (95%CI)	Control (n=131)
G241R	G	160 (72.7 )	0.29	0.84 (0.62-1.15)	202 (77.1)
	R	60 ( 27.3)			60 (22.9)
K469E	K	137 (62.3)	0.57	1.07(0.86-1.34)	156 (59.5)
	E	83 (37.7)			106 (40.5)

**Table 3:** Genotype frequencies of ICAM1 G241R and K469E polymorphisms of ICAM-1 gene in Iranian UC patients compared to healthy controls

SNP	Genotype	UC (n=110)	P-value	OR (95%CI)	Control (n=131)
	G/G	57 (51.8%)	1 (reference)		79 (60.3%)
G241R	G/R	46 (41.8%)	0.17	1.45 (0.85-2.48)	44 (33.6%)
	R/R	7 (6.4%)	0.72	1.21 (0.42-3.5)	8 (6.1%)
	K/K	45 (40.9%)	1 (reference)		42 (32.1%)
K469E	K/E	47 (42.7%)	0.82	0.6 (0.35-1.07)	72 (55.0%)
	E/E	18 (16.4%)	0.98	0.99 (0.35 -2.17)	17 (13.0% )

The frequencies were analyzed in UC and CD patients compared to controls separately, using the standard* χ*2 test. The SPSS software (version 17, USA) was utilized to perform the statistical analyses. Odds ratio (OR) were given with 95% confidence intervals, and p*≤*0.05 was considered significant. 

## Results

The mean age for UC and CD patients and controls were 36.5±12.8 (ranged from 16 to 65), 34.8±11.6 (ranged from 16 to 62), and 35.7±14.0 (ranged from 16 to 65) years, respectively. Patients and controls were matched for age and sex, 49.4% of each study group (UC, CD and control) were men and 50.6% were women.

Considering K469E polymorphism, the mutant allele of K469E polymorphism (E) was significantly more frequent in CD patients compared to controls (P< 0.05, OR= 1.83; 95% CI: 1.13 to 2.96) (Table 1). None of these two polymorphisms demonstrated any significant differences in allele or genotype frequencies in UC patients compared to controls (Table 2, 3). The homozygote mutant genotype (E/E) was also significantly more frequent in CD patients compared to healthy controls (P < 0.05, OR= 4.23; 95% CI: 1.42 to 12.59) (Table 4). The distributions of genotypes of ICAM-1 gene mutations, G241R and K469E, are demonstrated in Table 3 and 4.

**Table 4:** Genotype frequencies of ICAM1 G241R and K469E polymorphisms of ICAM-1 gene in Iranian CD patients compared to healthy controls

SNP	Genotype	CD(n=46)	P-value	OR (95%CI)	Control (n=131)
	G/G	20 (43.5%)	1 (reference)		79 (60.3%)
G241R	G/R	21 (45.7%)	0.08	1.89 (0.92-3.85)	44 (33.6%)
	R/R	5 (10.9%)	0.15	2.47 (0.73-8.37)	8 (6.1%)
	K/K	7 (15.2%)	1 (reference)		42 (32.1%)
K469E	K/E	27 (58.7%)	0.08	2.25 (0.90-5.61)	72 (55.0%)
	E/E	12 (26.1%)	0.009*	4.23 (1.42-12.60)	17 (13.0% )

**Table 5:** Genotype frequencies of G241R and K469E polymorphisms of ICAM1 gene in Iranian CD patients compared to UC patients

SNP	Genotype	CD(n=46)	UC (n=110)	P-value	OR (95%CI)
	G/G	20 (43.5%)	57 (51.8%)	1 (reference)	
G241R	G/R	21 (45.7%)	46 (41.8%)	0.47	1.3 (0.63-2.69)
	R/R	5 (10.9%)	7 (6.4%)	0.27	2.04 (0.58-7.14)
	K/K	7 (15.2%)	45 (40.9%)	1 (reference)	
K469E	K/E	27 (58.7%)	47 (42.7%)	0.006*	3.37 (1.46-9.32)
	E/E	12 (26.1%)	18 (16.4%)	0.008**	4.29(1.45-12.6)

We also compared the frequency of G241R and K469E polymorphisms between UC and CD patients (results are demonstrated in table 5). 

The mutant (E/E) genotypes of K469E had a statistically significant higher frequency in CD patients compared to UC patients (p< 0.05, OR= 0.37, 95% CI: 1.46-9.33). The K469E mutant allele frequency was also significantly higher in CD patients compared to UC patients (P< 0.05, OR= 2.05; 95% CI: 1.25-3.36). 

Among our patients, fibro-stenotic pattern was observed in two patients, who had mutant homozygote genotype for SNP K469E (E) and a mutant homozygote (R/R) and the heterozygote (G/R) genotype for SNP G241R of ICAM-1 gene.

We did not find any difference in the frequencies of G241R alleles and genotypes in CD or UC patients compared to controls (Data is shown in tables 1, 2). 

## Discussion

In our study, the mutant allele (E) of K469E polymorphism was more frequent in Crohn's patients compared to healthy controls (55.4% in CD vs. 40.5% in controls, P< 0.05, OR= 1.83; 95% CI= 1.13 to 2.96).

Previous studies in European population elucidated an association between the mutant allele of K469E (E) and various features of Crohn's disease, including fistulizing, and extensive disease, as well as disease location. However, none of these studies discovered a significant association of the K469E allele with disease susceptibility (2, 7, 8). The mutant genotype of K469E polymorphism (E/E) was significantly more frequent in Iranian patients with CD compared to controls (26.1% in CD patients vs. 13% in controls, P<0.05, OR=4.23, 95% CI: 1.42 -12.59). In a German study, the frequency of E/E genotype was 26% in CD patients vs. 9% in healthy controls (P= 0.002), which was very close to our results (10). Similar frequencies were observed in Italian population where the frequency of this allele was 24% in CD patients compared to 11.8% in controls (P< 0.001) (7, 8). 

In a meta-analysis study in Europe on 8 studies, including 801 CD, 627 UC patients, and 1828 controls, no association between CD and E469 allele were reported. However, an association was observed between the E469 allele and UC (P= 0.042, OR=1.425, 95% CI=1.01-2.00). The E/E genotype of the same polymorphism was also reported to be significantly more frequent in UC patients compared to controls (p=0.039, OR=2.054, 95% CI: 1.034-4.07). (16)

On the contrary, two independent studies in the United Kingdom and Japan, reported a lower frequency for E469 allele in IBD patients compared to controls (2, 9).

Another meta-analysis was performed in 2014 on 9 separate studies, including 1944 IBD (1029 CD and 915 UC) patients and 1316 control subjects. No significant association was reported between K469E polymorphism and IBD in the pooled data analysis. Only in one study, including the East Asian population, the E469 allele was reported to be more frequent in IBD patients compared to controls, which is similar to our study results (17).

The K469E polymorphism alters the production of soluble forms of ICAM-1, and modifies inflammatory immune responses by affecting cell–cell interactions. Thus, it may alter the function of this protein (18). Functional studies seem necessary to define the biological significance of the amino acid substitution resulting from the K469E polymorphism in ICAM-1 protein (2).

The frequency of K469E polymorphism varied significantly in the background population ranges from 9% (Germany) to 24.5% (United Kingdom) in Europe, while a low frequency was reported in Turkey (2.8%). In contrast, high frequency of this allele was observed in Japan (27.8%). The frequency of the mutant allele (E) in our healthy population was 40.5%, which was higher than previously mentioned studies.

This difference could partly explain the various associations observed in above-mentioned studies. Maybe other factors participate in the function of ICAM-1 protein and influence on the role of K469E polymorphism. The difference in this mutation frequency could be explained somehow with the genetic background of the studied populations.

The mutant allele of G241R polymorphism (R) was more frequent in Iranian CD and UC patients (33.7% and 27.3%, respectively) compared to healthy controls (22.9%), thus the difference was not statistically significant. There were no significant differences in the genotype frequencies of G241R polymorphism among IBD patients compared to controls either. Similar results were reported in Caucasian populations in the United States (19). In two studies in Turkey and Japan, no mutant allele (R241) was observed in the study population, including CD and UC, as well as controls (9, 15). 

In contrast to our results, the mutant allele (R) of G241R was found to be associated with UC in a study of a German population (16% in UC patients vs. 9% in controls, P=0.024) (7). However, other studies in Europe did not confirm the same results. 

In a study in the UK, though no significant difference was observed between these allele frequencies in IBD patients compared to controls; there was an association between limited disease extent and G/G genotype of G241R polymorphism in UC patients.

Considering the background population frequency of R241 allele, there is a wide range reported among various populations. Frequency of R241 allele is more prevalent in Europe and North America (15%), lower in Mediterranean population and absent in Japanese and Turkish populations (1, 15, 20-26).

The mutant allele (R) frequency of G241R, in our healthy population was 22.9%, which is closer to reports from Europe and North America. In conclusion, the present study demonstrated an overall association between the mutant allele (E) of K469E polymorphism and Crohn’s disease in Iranian population. Considering various frequencies and different above mentioned associations of ICAM-1 polymorphisms with IBD, further physio-pathological studies seem necessary to clear the precise function of this polymorphism in disease pathogenesis.

## Note

**(**Please cite as:** Habibi M, Naderi N, Farnood A, Balaii H, Dadaei T, Almasi SH, et al. Association between two single base polymorphisms of intercellular adhesion molecule 1gene, and inflammatory bowel disease. Gastroenterol Hepatol ****Bed Bench**** 2016;9(2):87-93).**
